# Serum Enzyme Profiles Differentiate Five Types of Muscular Dystrophy

**DOI:** 10.1155/2015/543282

**Published:** 2015-04-29

**Authors:** Yuling Zhu, Huili Zhang, Yiming Sun, Yaqin Li, Langhui Deng, Xingxuan Wen, Huaqiao Wang, Cheng Zhang

**Affiliations:** ^1^Department of Anatomy and Neurobiology, Zhongshan School of Medicine, Sun Yat-sen University, No. 58 Zhongshan 2nd Road, Guangzhou 510080, China; ^2^Department of Neurology, The First Affiliated Hospital, Sun Yat-sen University, No. 58 Zhongshan 2nd Road, Guangzhou 510080, China; ^3^Department of Healthcare Clinic, The First Affiliated Hospital, Sun Yat-sen University, No. 58 Zhongshan 2nd Road, Guangzhou 510080, China; ^4^Department of Laboratory Medicine, The First Affiliated Hospital, Sun Yat-sen University, No. 58 Zhongshan 2nd Road, Guangzhou 510080, China; ^5^Department of Epidemiology and Health Statistics, School of Public Health, Sun Yat-sen University, No. 58 Zhongshan 2nd Road, Guangzhou 510080, China

## Abstract

*Background.* Differentiation among types of muscular dystrophy (MD) has remained challenging. In this retrospective study, we sought to develop a methodology for differentiation of MD types using analysis of serum enzyme profiles.* Methods.* The serum levels of enzymes from 232 patients, including 120 with DMD, 36 with BMD, 36 with FSHD, 46 with LGMD, and 11 with EDMD, were evaluated.* Results.* The characteristic profiles of serum enzymes facilitated differentiation of these five types of MD. DMD was characterized by simultaneous elevation of ALT, AST, LDH, and ALP; BMD and LGMD were characterized by elevation of ALT, AST, and LDH; and FSHD and EDMD were characterized by a lack of abnormal serum enzyme levels. We further developed discriminant functions to distinguish BMD and LGMD. For LGMD, LGMD2B patients had significantly higher ALP levels than non-LGMD2B patients (98 ± 59 U/L versus 45 ± 9 U/L, resp., *p* < 0.05).* Conclusions.* Our approach enabled the determination of MD subtypes using serum enzyme profiles prior to genetic testing, which will increase the chance a mutation will be found in the first gene analyzed.

## 1. Introduction

Muscular dystrophies (MDs) are a heterogeneous group of inherited myopathies that share similar clinical features and dystrophic changes on muscle biopsies [1]. Despite the well-known disease symptoms, the diagnosis of MD continues to be challenging in the general pediatric settings and in pediatric neurology units [2, 3], potentially because unsuspected myopathy in children with hypertransaminasemia can be erroneously attributed to liver disease [4–11].

Alanine aminotransferase (ALT), aspartate aminotransferase (AST), alkaline phosphatase (ALP), and lactic dehydrogenase (LDH) are components of routine or comprehensive blood panels and collectively demonstrate liver function. Consequently, in apparently healthy children, analysis of these liver enzymes is performed more frequently than analysis of creatine kinase (CK), a more specific marker of muscle disease [12]. Particularly in rural or underdeveloped areas, a child with isolated hypertransaminasemia, labeled as being affected by cryptogenic hepatopathy, could be monitored only with liver function tests for a long time before serum CK is analyzed or before a muscular disease becomes clinically obvious, thus delaying diagnosis and treatment [13].

With advancements in diagnostic methodologies, such as magnetic resonance imaging (MRI), muscle biopsy, and genetic screening, more types of MD can be categorized accurately. However, because not all hospitals have access to these advanced techniques, the diagnosis of MD can still be challenging. CK values may facilitate differential diagnosis to some extent [13], but measurement of CK alone is not as comprehensive as measurement of other serum enzymes. Thus, the development of additional tools for serum enzymes tests may facilitate differential diagnosis of subtypes of MD.

Therefore, in this study, we retrospectively reviewed the clinical records of hundreds of Chinese patients with MD, in order to examine changes in enzyme profiles in different types of MD. Our results emphasize that a diagnosis of occult muscle disease should be considered when confronted with an unexplained elevation of serum enzymes.

## 2. Patients and Methods

### 2.1. Patients

Clinical data from Chinese patients with MD who visited the Department of Neurology at the First Affiliated Hospital of Sun Yat-sen University were collected between June 2012 and October 2013. Patients were excluded if they had any coexisting medical diseases according to medical records. This study was approved by the Local Ethical Committee at Sun Yat-sen University (China) and was conducted in accordance with the recommendations of the Declaration of Helsinki. Adult patients and parents of affected children provided written informed consent.

Patients had been diagnosed with one of the following five pathologies: (1) Duchenne muscular dystrophy, (2) Becker's muscular dystrophy (BMD), (3) facioscapulohumeral dystrophy (FSHD), (4) limb girdle muscular dystrophy (LGMD), or (5) Emery-Dreifuss muscular dystrophy (EDMD). For DMD/BMD, patients were diagnosed by dystrophin gene analysis or immunohistochemistry and western blotting for dystrophin on muscular biopsy specimen. FSHD was confirmed by two neurologists on the basis of clinical manifestation. LGMD was identified according to traditional clinical, electrophysiological, and histological criteria, and diagnoses of DMD/BMD, FSHD, polymyositis, and myotonic dystrophy were excluded simultaneously. Some cases of LGMD were confirmed by gene analysis. EDMD was diagnosed according to previously published criteria [14], and some of cases were confirmed by gene analysis.

### 2.2. Laboratory Measurements

Serum enzymes, including ALT, AST, ALP, LDH, and CK, were measured using an Abbott Aeroset fully automatic biochemical analyzer (Abbott Laboratories, USA). The levels of serum enzymes were assayed according to the instructions provided with the corresponding enzymatic kits. The upper limits of normal for ALT, AST, ALP, LHD, and CK were 40, 37, 110, 240, and 250 U/L, respectively.

### 2.3. Statistical Analysis

Statistical analysis was performed using SPSS, Version 20.0 (IBM SPSS Statistics 20.0). Due to the differences in the normal ranges of different enzymes, new variables were adopted appropriately for analysis; variables ALTn, ASTn, ALPn, and LDHn were defined as the value of the enzyme divided by the upper limit of normal (ULN) for that enzyme. The normal distributions of the variables were tested with the Kolmogorov-Smirnov test (*n* > 50) or Shapiro-Wilk test (*n* ≤ 50). Variables (distributed normally) were reported as the mean and standard deviation (mean ± SD) or as the median and the 25th and 75th percentiles. Since not all variables were distributed normally, Mann-Whitney rank sum tests and Bonferroni tests (*a*′ = 0.005) were applied to compare the enzyme values of one group with another. We then performed one-sample *t*-tests or rank sum tests to determine magnitude of changes in enzyme levels. To distinguish between the BMD and LGMD groups, discrimination analysis was performed to develop discriminant functions derived from the Fisher principle. ALT, AST, ALP, LDH, CK, age, and gender were regarded as independent variables, and category of diagnosis (BMD or LGMD) was regarded as the dependent variable. All tests were two-tailed, and differences with *p* values of less than 0.05 were considered statistically significant.

## 3. Results

### 3.1. Patient Demographic Characteristics

A total of 232 patients were included in this study, of which 120 patients were diagnosed with DMD, 36 patients were diagnosed with BMD, 19 were diagnosed with FSHD, 46 were diagnosed with LGMD, and 11 were diagnosed with EDMD. The mean age of patients with DMD was the lowest of all subtypes (~7 years), while the mean age of patients with FSHD was the highest of all subtypes (~26 years). More than 97% of patients with DMD and BMD had abnormal ALT, AST, and LDH values. The proportion of patients with abnormal ALT and AST values was lowest in patients with EDMD (27.3% and 36.4%, resp.). More than 40% of patients with FSHD and LGMD had abnormal ALT, AST, ALP, and LDH values, with the exception of AST in FSHD, which was abnormal in less than 40% of patients (36.8%). The demographics and frequencies of patients with MD presenting with abnormal serum enzymes are shown in Table 1.

### 3.2. Serum Enzyme Levels among Five Types of MD

For ALT, AST, and LDH levels, patients with DMD had higher serum concentrations than patients with BMD, FSHD, LGMD, and EDMD (*p* < 0.05), and patients with BMD had higher serum concentrations than patients with FSHD, LGMD, and EDMD (*p* < 0.05). In addition, patients with LGMD had higher ALTn concentrations than patients with EDMD (*p* < 0.05). However, ALTn concentrations did not differ between patients with FSHD and LGMD or between patients with FSHD and EDMD. In contrast, patients with FSHD had significantly lower ASTn concentrations than patients with LGMD (*p* < 0.05). Similarly, patients with LGMD had higher ALTn concentrations than patients with EDMD (*p* < 0.05). However, no differences in LDH concentrations were observed between these groups.

Serum ALP profiles were different from those of the other three enzymes (ALT, AST, and LDH). Significant differences were only observed between patients with DMD and FSHD and between patients with DMD and LGMD (*p* < 0.05). Additionally, in patients with DMD, BMD, FSDH, and LGMD, the fold changes for ALT, AST, and LDH were greater than that for ALP, while, in patients with EDMD, the fold increases for ALT, AST, and LDH were lower than that for ALP. Serum enzymes concentrations in patients with the five different types of MD are shown in Table 2.

### 3.3. Profiles of Serum Enzymes in Different Types of MD

For patients with different types of MD, only patients with DMD exhibited simultaneous elevation of serum ALT, AST, ALP, and LDH values. ALT levels exhibited the greatest increase, followed by AST, LDH, and ALP (Table 3).

Elevated serum ALT, AST, and LDH values were observed in patients with BMD or LGMD (*p* < 0.05), while serum ALP values remained within the normal range (*p* > 0.05). However, these two MD subtypes could be roughly distinguished by the magnitudes of changes in these enzymes (Table 4). For patients with BMD, ALT and AST levels exceeded 2-fold the ULN. In contrast, in patients with LGMD, ALT and AST levels were about 2-fold the ULN. Since LDH levels in patients with BMD and LGMD were both 2-fold the ULN (*p* > 0.05 compared with “2”), we did not detect significant differences in LDH levels between patients with these subtypes. However, patients with BMD tended to have higher serum LDH levels. In patients with BMD, ALTn and ASTn levels exhibited the greatest increase, followed by LDHn and ALPn. In contrast, in patients with LGMD, ALTn, ASTn, and LDHn levels exhibited similar increases, while the increase in ALPn was lower.

Patients with FSHD and EDMD exhibited the same profiles for the four liver enzymes, with all values remaining within the normal range (Table 3). Nonetheless, patients with FSHD tended to have higher serum ALT and AST values than patients with EDMD, whereas patients with EDMD tended to have higher serum ALP and LDH values than patients with FSHD (*p* > 0.05).

### 3.4. Discriminant Functions to Identify Patients with BMD and LDMD

Because age and gender were correlated with diagnosis, we developed discriminant functions to distinguish between BMD and LGMD more accurately without gene detection or muscle biopsy (Table 5). Once serum enzyme levels (ALT, AST, ALP, and LDH) were measured in patients of known age, we could separately calculate two mathematical values (*Y*_1_ and *Y*_2_), and by comparison of *Y*_1_ and *Y*_2_, clinicians could make a probable diagnosis of BMD when *Y*_1_ was greater than *Y*_2_. On the contrary, a probable diagnosis of LGMD could be established when *Y*_2_ was greater than *Y*_1_.

### 3.5. Serum Enzyme Levels in Patients with LGMD

Twenty-six patients with LGMD (over 50%) had genetic tests. Among them, five had duplicate genetic tests (data not shown). Three patients carried* CAPN3* gene mutations responsible for LGMD2A, and three patients were negative for the* CAPN3* gene mutation. Moreover, eight patients harbored* DYSF* gene mutations responsible for LGMD2B, and eight patients were negative for the* DYSF* gene mutation.

Since there were few samples from patients with LGMD2A, we could not perform any statistical analysis to detect characteristic profiles of serum enzymes between patients with and without LGMD2A. There were no significant differences in ALT, AST, or LDH levels between patients with and without LGMD2B (data not shown). Additionally, no significant differences in CK levels were observed between patients with and without LGMD2B. However, ALP levels in LGMD2B patients (98 ± 59 U/L) were significantly higher than those in non-LGMD2B patients (45 ± 9 U/L; *t* = −2.474, *p* = 0.04).

## 4. Discussion

Here, we presented a retrospective analysis of serum enzyme levels from patients with MD, who were originally diagnosed according to clinical and/or genetic diagnoses. Although serum CK testing is an easy, sensitive, and inexpensive test for muscular diseases, it appears to be underutilized in routine clinical practice. In apparently healthy children, aminotransferase levels are assessed more frequently than CK or aldolase levels [15]. Economic restrictions may negatively influence the broadening of laboratory tests [16], and transaminases may be the only targets analyzed before signs of a muscular disease become clinically obvious in patients with incidental elevations in aminotransferase levels. Ciafaloni et al. reported that initial evaluations in children presenting with motor or global developmental delays included CK screening in only 35% of cases [3]. Hence, routine biochemical assays, such as transaminases, ALP, LDH, and CK analyses, should be evaluated for their predictive ability.

In the present study, a high frequency of patients with MD presented with abnormal levels of serum enzymes (including ALT, AST, ALP, and LDH). For instance, all patients with BMD and up to 97% of patients with DMD had elevated ALT, AST, and LDH values. Even in patients with EDMD, for which the frequency was relatively small, the proportion of patients presenting with abnormal ALT, AST, or LDH values was no lower than 25%. Indeed, studies from around the world [4, 6–8, 11, 15, 17] have reported that patients with muscular diseases are often erroneously labeled as having cryptogenic liver disease. However, most of these studies have analyzed aminotransferases, with few analyses of ALP and LDH. Hence, serum ALP and/or LDH as markers of muscle diseases should also be stressed.

The mechanism through which levels of ALP and LDH become abnormal in patients with MD is still unknown. Elevations in ALT and AST levels are common indicators of hepatocellular damage; however, ALT abnormalities are also found in cardiac and skeletal muscle, although ALT activity in skeletal muscle is only one-tenth of that in hepatocytes [18]. AST is found within the cardiac muscle, skeletal muscle, kidneys, brain, pancreas, lungs, leukocytes, and erythrocytes [17]. Since serum CK is markedly elevated with breakdown of muscle and is considered a diagnostic marker of MDs [19], we assumed that leakage of transaminases from muscle membrane would occur along with the leakage of CK under pathological conditions, such as in patients with MD.

Serum enzyme levels were elevated to variable degrees among patients with different subtypes of MD. McMillan et al. reported that ALT values are elevated by up to 22.6 times the ULN in patients with DMD [20], whereas we observed that ALT reached 5–8 times the ULN in patients with DMD. Different methods for detecting enzyme levels or taking blood samples under nonstandardized conditions may account for this discrepancy. Additionally, Zhang et al. reported that disorders can be sequenced (e.g., DMD/BMD > LGMD > FSHD) according to AST or ALT levels [21], consistent with our results. Regarding LDH, Yasmineh et al. reported that the mean total serum activity in patients with DMD was 3.4-fold that of serum from the control group [22], which was consistent with the range observed in our current analysis (3.23 to 5.60). Our observations were also consistent with previous reports demonstrating that serum LDH activity in patients with EDMD was within the normal limit or slightly increased [14, 23]. As another index, ALP levels have seldom been described in muscular disease. Strikingly, in patients with DMD, BMD, FSDH, and LGMD, the fold increases for ALT, AST, and LDH were greater than that for ALP, while in patients with EDMD, the fold increases for ALT, AST, and LDH were lower than that for ALP. Further research is needed to determine the correlation between EDMD and ALP.

To some extent, CK values may facilitate differential diagnosis [24]. Hence, when advanced diagnostic technology is absent, discrepancies in levels of other enzymes may also be used to provide important clues. Interestingly, each type of MD had a characteristic profile of serum enzymes. Therefore, the distribution of serum enzymes may have additional implications for the differential diagnosis of MD. Moreover, although patients with FSHD and EDMD shared the same serum enzyme profiles, distinguishing between these diseases is relatively simple based on clinical features alone. For example, FSHD is characterized by weakness of the face, upper-arm, and shoulder girdle muscles [24], whereas EDMD is characterized by slow progressive muscle weakness, early joint contractures, and atrial arrhythmias [25]. Thus, differential diagnoses should consider as many parameters as possible.

Clinical differential diagnosis between BMD and LGMD may be difficult because the clinical phenotype of BMD tends to overlap with other limb girdle syndromes, especially LGMD [26]. In fact, in our tertiary care center, we continue to observe misdiagnosis of BMD as LGMD and vice versa. Genetic analysis is the gold standard for distinguishing between these disorders. However, it is difficult to perform genetic analysis when first evaluating a patient suspected of LGMD because of the various subtypes of LGMD. Hence, additional methods for distinguishing between BMD and LGMD, as well as subtypes of LGMD, should be developed. As illustrated here, serum enzyme levels were elevated to variable degrees in patients with BMD or LGMD. For the former, ALT or AST levels were more than 2-fold the ULN, and, for the latter, ALT or AST levels were equal to 2-fold the ULN. In addition, we provided discriminant functions to assist clinicians in identification of these subtypes without advanced diagnostic technology as follows: once serum enzyme levels (ALT, AST, ALP, and LDH) are measured in patients of known age, clinicians can make a probable diagnosis of BMD when *Y*_1_ is greater than *Y*_2_ or of LGMD when *Y*_2_ is greater than *Y*_1_.

LGMD is a heterogeneous genetically determined group of skeletal muscle disorders. Because at least 18 genetically distinct subtypes of LGMD have been described, determining the exact subtype of LGMD in a particular patient can be challenging [27]. In our study, over half of patients with LGMD had genetic tests. However, only three patients were positive for* CAPN3* gene mutations, and eight patients were positive for* DYSF* gene mutations. This low positive rate necessitates the urgent detection of appropriate clinical information to narrow the scope of gene testing. The CK value also serves as an important clue to facilitate the differential diagnosis of subtypes. For example, a marked elevation in CK concentrations has been shown to occur in confirmed cases of LGMD2B [28]. However, we failed to detect significant differences in CK concentrations between patients with and without LGMD2B, which may be explained by the limited number of cases analyzed here. Moreover, higher ALP levels were observed in patients with LGMD2B, suggesting another parameter for distinguishing LGMD2B from other types of LGMD in clinical practice. Therefore, further studies of LGMD cases confirmed by genetic test are needed to determine the correlations between genotype and serum enzyme levels.

Our study had several limitations. First, only 11 cases of EDMD and 19 cases of FSHD were reviewed in the present study. It is likely that the small sample studied is not representative of the general patient population. Second, not all the patients with LGMD were confirmed by genetic testing, and the exact diagnosis of LGMD subtypes was challenging. Third, we did not thoroughly investigate the effects of some medications or food on serum enzyme levels. Marked variability in serum enzymes can occur from day to day [29].

In summary, we found that a high frequency of patients with MD presented with abnormal serum enzyme levels. The characteristic profiles of serum enzymes facilitated the differentiation of MD subtypes. For example, DMD was characterized by simultaneous elevation of ALT, AST, LDH, and ALP; BMD and LGMD were characterized by elevation of ALT, AST, and LDH; and FSHD and EDMD lacked abnormalities in the serum levels of these four enzymes. To further differentiate BMD from LGMD, discriminant functions were developed for cases in which enzyme levels and age are known. For LGMD, patients with LGMD2B had significantly higher ALP levels than patients with non-LGMD2B subtypes. Thus, our approach makes it possible to determine the subtypes of MD by serum enzyme profiles prior to genetic testing, which will increase the chance that a mutation will be found in the first gene analyzed.

## Figures and Tables

**Table 1:** The demography and frequency of patients with MD presented with normal or abnormal serum enzymes levels.

Category	Age (y)	Gender *n* (%)		ALTn *n* (%)		ASTn *n* (%)		ALPn *n* (%)		LDHn *n* (%)		CKn *n* (%)	
	Mean ± SD (range)	Male	Female	Normal	Abnormal	Normal	Abnormal	Normal	Abnormal	Normal	Abnormal	Normal	Abnormal
DMD	6.7 ± 3.4 (7 m–24)	120 (100)	0 (0)	3 (2.5)	119 (97.5)	3 (2.5)	119 (97.5)	39 (32)	83 (68)	3 (2.5)	119 (97.5)	0 (0)	122 (100)
BMD	13.0 ± 8.5 (2–37)	36 (100)	0 (0)	0 (0)	37 (100)	0 (0)	37 (100)	16 (43.2)	21 (56.8)	0 (0)	37 (100)	0 (0)	37 (100)
FSHD	25.8 ± 9.3 (13–50)	13 (68)	6 (32)	5 (26.3)	14 (73.7)	12 (63.2)	7 (36.8)	10 (52.6)	9 (47.4)	11 (57.9)	8 (42.1)	1 (5.3)	18 (94.7)
LGMD	22.6 ± 11.4 (3–54)	23 (56)	18 (44)	11 (26.8)	30 (73.2)	9 (22.0)	32 (78.0)	24 (58.5)	17 (41.5)	10 (24.4)	31 (75.6)	1 (2.4)	40 (97.6)
EDMD	15.4 ± 9.9 (6–42)	6 (55)	5 (45)	8 (72.7)	3 (27.3)	7 (63.6)	4 (36.4)	4 (36.4)	7 (63.6)	6 (54.5)	5 (45.5)	3 (27.3)	8 (72.7)

MD, muscular dystrophy; DMD, Duchenne muscular dystrophy; BMD, Becker's muscular dystrophy; FSHD, facioscapulohumeral dystrophy; LGMD, limb girdle muscular dystrophy; EDMD, Emery-Dreifuss muscular dystrophy; ALT, alanine aminotransferase; AST, aspartate aminotransferase; ALP, alkaline phosphatase; LDH, lactic dehydrogenase; CK, creatine kinase; m, month.

**Table 2:** Serum enzymes levels among five types of MD.

Category	ALTn (*P*_25_–*P*_75_)	ASTn (*P*_25_–*P*_75_)	ALPn (*P*_25_–*P*_75_)	LDHn (*P*_25_–*P*_75_)	CKn (*P*_25_–*P*_75_)
DMD	6.55 (4.85–8.18)^b,c,d,e^	5.32 (3.98–6.68)^b,c,d,e^	1.1 (0.94–1.25)^c,d^	4.28 (3.23–5.60)^b,c,d,e^	44.77 (31.05–56.57)^b,c,d,e^
BMD	2.91 (1.96–4.61)^a,c,d,e^	2.85 (1.76–4.5)^a,c,d,e^	1.06 ± 0.48	2.02 (1.49–3.48)^a,c,d,e^	27.59 (16.64–41.22)^a,c,d,e^
FSHD	1.04 ± 0.48^a,b^	1.00 (0.87–1.44)^a,b,d^	0.60 (0.42–0.92)^a^	1.02 (0.93–1.44)^a,b^	3.05 (1.64–3.91)^a,b,d^
LGMD	1.65 (0.74–3.39)^a,b,e^	1.68 (1.01–2.86)^a,b,c,e^	0.55 (0.45–1.17)^a^	1.49 (1.00–2.37)^a,b^	9.08 (4.50–21.87)^a,b,c,e^
EDMD	0.50 (0.36–0.83)^a,b,d^	0.73 (0.69–1.04)^a,b,d^	1.15 ± 0.54	1.09 ± 0.17^a,b^	1.64 (0.66–3.51)^a,b,d^
*H *	92.45	111.09	22.83	112.19	114.01
*p* value	<0.001	<0.001	<0.001	<0.001	<0.001

MD, muscular dystrophy; DMD, Duchenne muscular dystrophy; BMD, Becker's muscular dystrophy; FSHD, facioscapulohumeral dystrophy; LGMD, limb girdle muscular dystrophy; EDMD, Emery-Dreifuss muscular dystrophy; ALT, alanine aminotransferase; AST, aspartate aminotransferase; ALP, alkaline phosphatase; LDH, lactic dehydrogenase; CK, creatine kinase.

^a^Comparing with DMD, *p* < 0.05.

^b^Comparing with BMD, *p* < 0.05.

^c^Comparing with FSHD, *p* < 0.05.

^d^Comparing with LGMD, *p* < 0.05.

^e^Comparing with EDMD, *p* < 0.05.

**Table 3:** The serum enzymes profile in five types of MD.

Category	ALTn (*P*_25_–*P*_75_)	ASTn (*P*_25_–*P*_75_)	ALPn (*P*_25_–*P*_75_)	LDHn (*P*_25_–*P*_75_)
BMD	2.91 (1.96–4.61) (↑)	2.85 (1.76–4.5) (↑)	1.06 ± 0.46 (→)	2.02 (1.49–3.48) (↑)
DMD	6.55 (4.85–8.18) (↑)	5.32 (3.98–6.68) (↑)	1.1 (0.94–1.25) (↑)	4.28 (3.23–5.60) (↑)
EDMD	0.50 (0.36–0.83) (→)	0.73 (0.69–1.04) (→)	1.15 ± 0.54 (→)	1.06 ± 0.19 (→)
FSHD	1.04 ± 0.48 (→)	1.00 (0.87–1.44) (→)	0.60 (0.42–0.92) (→)	1.02 (0.93–1.44) (→)
LGMD	1.65 (0.74–3.39) (↑)	1.68 (1.01–2.86) (↑)	0.55 (0.45–1.17) (→)	1.49 (1.00–2.37) (↑)

MD, muscular dystrophy; DMD, Duchenne muscular dystrophy; BMD, Becker's muscular dystrophy; FSHD, facioscapulohumeral dystrophy; LGMD, limb girdle muscular dystrophy; EDMD, Emery-Dreifuss muscular dystrophy; ALT, alanine aminotransferase; AST, aspartate aminotransferase; ALP, alkaline phosphatase; LDH, lactic dehydrogenase.

“→” represents that patients had similar levels of enzymes comparing with normal people (*p* > 0.05). “↑” represents that patients had significantly higher levels of enzymes comparing with normal people (*p* < 0.05).

**Table 4:** The enzymes profile between patients with BMD and LGMD.

Category	ALTn (*P*_25_–*P*_75_)	ASTn (*P*_25_–*P*_75_)	ALPn (*P*_25_–*P*_75_)	LDHn (*P*_25_–*P*_75_)
BMD	2.91 (1.96–4.61) (↑)	2.85 (1.76–4.5) (↑)	1.06 ± 0.46	2.02 (1.49–3.48) (→)
LGMD	1.65 (0.74–3.39) (→)	1.68 (1.01–2.86) (→)	0.55 (0.45–1.17)	1.49 (1.00–2.37) (→)

BMD, Becker's muscular dystrophy; LGMD, limb girdle muscular dystrophy; ALT, alanine aminotransferase; AST, aspartate aminotransferase; ALP, alkaline phosphatase; LDH, lactic dehydrogenase.

“→” represents that patients had 2-fold ULN of enzymes levels (compared with “2,” *p* > 0.05). “↑” represents that patients had exceeded 2-fold ULN of enzymes levels (compared with “2,” *p* < 0.05). As demonstrated in Table 2, there was no significant difference of ALP serum levels in patients with BMD and LGMD, so we did not perform further analysis on magnitude of change in ALP serum levels.

**Table 5:** Discriminant functions to identify patients with BMD and LGMD.

Discriminant functions
*Y*_1_ = −19.406 + 0.724*X*_1_ − 0.003*X*_2_ + 0.032*X*_3_ + 0.80*X*_4_ + 0.001*X*_5_ + 13.299*X*_6_
*Y*_2_ = −30.537 + 0.909*X*_1_ *e* − 0.007*X*_2_ + 0.030*X*_3_ + 0.92*X*_4_ + 0.002*X*_5_ + 18.745*X*_6_

BMD, Becker's muscular dystrophy; LGMD, limb girdle muscular dystrophy; ALT, alanine aminotransferase; AST, aspartate aminotransferase; ALP, alkaline phosphatase; LDH, lactic dehydrogenase.

*X*_1_ is age, *X*_2_ is ALT, *X*_3_ is AST, *X*_4_ is ALP, *X*_5_ is LDH, and *X*_6_ is gender, *Y*_1_ represents a diagnosis of BMD, and *Y*_2_ represents a diagnosis of LGMD.
